# Studies on Insecticidal Activities and Action Mechanism of Novel Benzoylphenylurea Candidate NK-17

**DOI:** 10.1371/journal.pone.0066251

**Published:** 2013-06-11

**Authors:** Yongqiang Li, Yaoguo Qin, Na Yang, Yufeng Sun, Xinling Yang, Ranfeng Sun, Qingmin Wang, Yun Ling

**Affiliations:** 1 Key Laboratory of Pesticide Chemistry and Application, MOA, Department of Applied Chemistry, College of Science, China Agricultural University, Beijing, People’s Republic of China; 2 State Key Laboratory of Elemento-Organic Chemistry, Institute of Elemento-Organic Chemistry, Nankai University, Tianjin, People’s Republic of China; CNR, Italy

## Abstract

Insecticidal activity of **NK-17** was evaluated both in laboratory and in field. It was found that the toxicity of **NK-17** against *S. exigua* was 1.93 times and 2.69 times those of hexaflumuron and chlorfluazuron respectively, and the toxicity of **NK-17** against *P. xylostella* was 1.36 times and 1.90 times those of hexaflumuron and chlorfluazuron respectively, and the toxicity of **NK-17** against *M. separate* was 18.24 times those of hexaflumuron in laboratory, and 5% **NK-17** EC at 60 g a.i ha^−1^ can control *S. exigua* and *P. xylostella* with the best control efficiency of about 89% and over 88% respectively in Changsha and Tianjin in field. The insecticidal mechanism of **NK-17** was explored for the first time by utilizing the fluorescence polarization method. **NK-17** could bind to sulfonylurea receptor (SUR) of *B. germanica* with stronger affinity comparing to diflubenzuron and glibenclamide, which suggested that **NK-17** may also act on the site of SUR to inhibit the chitin synthesis in insect body and the result can well explain that **NK-17** exhibited stronger toxicity against *B. germanica* than diflubenzuron and glibenclamide *in vivo*.

## Introduction

**Figure 1:**
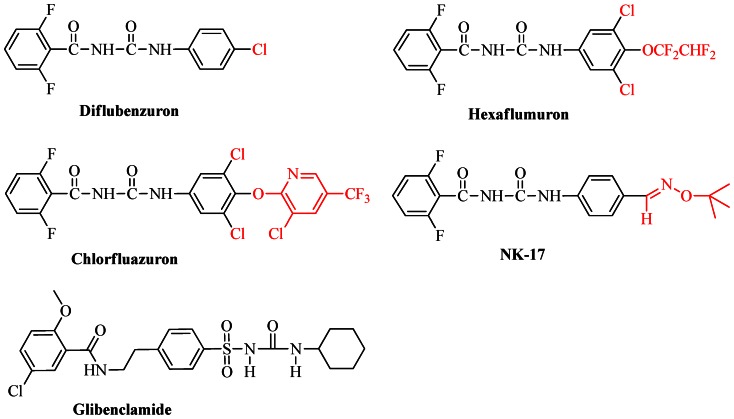
Chemical structures of benzoylphenylureas, NK-17 and glibenclamide.

The abundance of insects belonging to the order of Lepidoptera, such as *Mythimna separata*, *Plutella xylostella*, *Spodoptera exigua* and *Hyphantria cunea* etc., are one type of the most damaging pests for crops and forests. Because they may be able to cause significant damage in the process of agricultural production, a variety of insecticides were utilized to control the Lepidoptera pests since 1950s. The insecticides brought numerous benefits, meanwhile, they have negative effects such as environmental pollution, toxicity to nontarget organisms including mammals, and the insecticide resistance increased year by year. Therefore, researchers were pushed to develop novel, highly efficient, low toxicity, friendly environmental insecticides. Benzoylphenylureas (BPUs), acting on the larval stages of the Lepidoptera insects by inhibiting chitin synthesis as an important type of insect growth regulators (IGRs), have been rapidly developed since the first benzoylphenylurea (diflubenzuron, DFB, Figure 1) was introduced to the market in 1972 [1]. Besides diflubenzuron, hexaflumuron and chlorfluazuron (Figure 1) were some of other widely used insecticides. The 2000 “Presidential Green Chemistry Challenge” was awarded to Dow AgroSciences LLC for their innovation of Sentricon Termite Colony Elimination System, a new paradigm for termite control, which contained hexaflumuron as a major active ingredient [2]. Benzoylphenylureas have a unique mode of action coupled with a high degree of activity on target pests and low toxicity to nontarget organisms [3]–[4], thus have become a new tool for integrated pest management. Because of the above advantages, benzoylphenylureas have attracted considerable attention for decades [5]–[15]. In particular**,** we have designed and synthesized novel benzoylphenylureas containing oxime ether group, and found that these benzoylphenylureas exhibited excellent larvicidal activities against oriental armyworm and mosquito. For example, compound **NK-17** (Figure 1) exhibited excellent larvicidal activity against mosquito, which had 90% mortality even at 0.001 mg L^−1^ [16]–[17]. According to the comprehensive analysis of bioactivity, physical properties and synthetic procedure etc., we chose compound **NK-17** for further development as a novel and potent insecticide. It is very important that evaluation of insecticidal activities is a critical step to a new insecticide candidate with independent intellectual property right before it is introduced to the market. Hence, in this paper we will study insecticidal activities of **NK-17** in laboratory and in field to evaluate its application prospects.

The initial study results showed that chitin synthetase catalyzing the incorporation of UDP-*N*-acetylglucosamine (UDP-NAGA) to chitin was clearly inhibited by BPUs *in vivo* or *in situ* (isolated integument incubated in a tissue culture medium). However, all of the subsequent studies trying to prove some action of BPUs on any part of the chitin synthesis pathway in insects in *cell free* systems (rather than *in vivo* and *in situ*) failed [18]–[22]. In addition, BPUs showed no inhibitory actions on fungal chitin synthetase *in vivo* as well as in *cell free* systems, which have roughly equivalent chitin synthesis pathways as insects [18]. Therefore, the action mechanism of BPUs remained unresolved. In 2004, Matsumura and co-workers reported that the benzoylurea insecticide diflubenzuron acted on the same target site on the sulfonylurea receptor (SUR) protein as a typical sulfonylurea drug, glibenclamide, in *Drosophila melanogaster* and *Blattella germanica*. Furthermore, such action by these chemicals is the cause of their inhibitory effect on chitin synthesis [22]–[23]. It is a great discovery on the action mechanism research of benzoylurea insecticides.

In recent years, more evidences have been provided that the sulfonylurea receptor (SUR) protein, the action site of DFB, attributing to the ATP-binding cassette (ABC) transporter superfamily proteins [24]. The ABC proteins execute a number of important physiological functions in the biosystems such as chemical exchange, ion channels, receptors, and signal transduction etc.. SUR associates with four pore-forming (Kir6.x) potassium channel subunits (4 SUR subunits and 4 Kir6.x subunits) to form ATP-sensitive potassium channels(K_ATP_). Some researches suggested that the Kir6.x subunits, which constitute the pore of K_ATP_, are located centrally, whereas the SUR subunits are located peripherally [25]–[27]. The K_ATP_ has a unique function among the ABC superfamily of proteins, because the Kir6.x subunits manage transport of potassium, however, the SUR subunits play are regulatory role modulating channel activity. ATP can bind to the SUR subunits to inhibit K_ATP_ channel activity and regulate a series of biochemical systems, similarly, sulfonylureas, which are widely used in the treatment of type 2 diabetes mellitus as a ligand of SUR, bind to the SUR subunit in multiple locations so as to inhibit K_ATP_ channel activity too.

However, above research about SUR proteins involve in utilizing the isotope labeling technology. Due to the restriction by the level of laboratory condition and safety to the researchers, the isotope labeling technology cannot be quickly and easily used in usual laboratory.

The fluorescence polarization (FP) assay is based on the high affinity binding of the fluorescence probe N-Phenyl-1-naphthylamine (1-NPN) to a specific site of SUR. After absorbing polarized light, free 1-NPN of relatively small molecular weight emits light in all directions due to the fast tumbling rate, resulting in low polarization. When binding to the target protein SUR, the 1-NPN molecules rotate slower due to the larger combined molecular size of the complex. Consequently, they emit radiation in the same direction as that of the incident light, and exhibit higher polarization. When some compounds can displace the 1-NPN molecules from SUR, the disruption of the binding between the 1-NPN molecules and the protein can be identified by decreased polarization. Thus, the FP assay can be used for determination of small molecule chemical ligand binding affinity to SUR in vitro. Hence, in this paper we will adopt FP method to study **NK-17** binding affinity to SUR comparing with glibenclamide and DFB and then obtain the effect-dose curves by dose-dependent validation experiments with these compounds, so ascertain the binding mechanism of diflubenzuron and glibenclamide to the SUR, and study the action mechanism of **NK-17** for the fisrt time.

## Materials and Methods

### Insects

Beet armyworm (*Spodoptera exigua*, *S*. *exigua* ) has been reared in the bioassay platform of State Key Laboratory of Elemento-Organic Chemistry, Nankai University since 2008 in the standard laboratory conditions of 27±1°C, 50%∼75% RH and under an Light : Dark (L:D) 14∶10 h photoperiod. Third-instar larvae were raised on the artificial diets and used in bioassay experiments.

Oriental armyworm (*Mythimna separate*, *M. separata*) has been introduced into the bioassay platform of State Key Laboratory of Elemento-Organic Chemistry, Nankai University in 1995 under a climatic chamber (25±1 °C, 60%∼70% RH and under an L:D 13∶11 h photoperiod). Fourth-instar larvae were raised on the artificial diets and used in bioassay experiments.

Diamondback moth (*Plutella xylostella*, *P*. *xylostella*) was raised on wild cabbage (Brassica oleracea L) in the greenhouse of the bioassay platform of State Key Laboratory of Elemento-Organic Chemistry, Nankai University plants under standard laboratory conditions (23±1°C, 50%∼60% RH and natural lighting). Third-instar larvae were used in bioassay experiments.

German cockroach (*Blattella germanica*, *B*. *germanica*) was fed dog food and water and maintained in cheesecloth-covered plastic, the rim of which was coated with Vaseline. The cockroach colony was maintained at 27±2°C, 60±5% relative humidity and 12∶12 h (L:D).

### Reagents and compounds

Chlorfluazuron (purity 98%) was purchased from Shijiazhuang Jitai Sanmu Pesticide Chemical Industry Co., Ltd. 5% chlorfluazuron EC and hexaflumuron (purity 95%) were purchased from Jiangsu Yangnong Chemical Group Co., Ltd. 5% hexaflumuron EC was purchased from Tianjin Shipule Pesticide Technical DEP. Co., Ltd. Tween-20 and dimethyl sulfoxide (DMSO) were purchased from Alfa Aesar China (Tianjin) Co., Ltd. **NK-17** (>99%) was synthesized according to our previously reported method [16]. 5% **NK-17** EC was prepared in our research group. All other biochemical reagents including glibenclamide and diflubenzuron were of the highest purity grade from Sigma-Aldrich Chemical Co., Ltd.

### Bioassay against *S. exigua* and *P. xylostella* in laboratory

The bioassay of **NK-17** and contrast compounds chlorfluazuron and hexaflumuron against the beet armyworm (*S. exigua*) and the diamondback moth (*P. xylostella*) were tested by the leaf-dip method. For each test sample, a stock solution at a concentration of 200 mg L^−1^ in DMSO was prepared and then diluted to the required series concentrations with water containing Tween-20. Leaf disks (5 cm×1 cm) from fresh cabbage leaves were dipped into the test solution for 10 s. After air-drying on a filter paper, the leaf disks were individually placed into Petri dishes (7 cm diameter). Third-instar larvae were individually transferred into the Petri dishes. Infested leaves treated with water and DMSO were provided as blank controls. Six replicates (10 larvae per replicate) were performed, and the results were expressed as mean value of replicates. Percentage mortalities were evaluated 72–96 hours after treatment in the culture conditions and corrected with Abbott’s formula [28]–[30]. When the percentage mortality of the control is less than 5%, the result is directly used. However, when the percentage mortality was less than 20%, the result was corrected by V  =  ((X–Y)/X)*100 (V  =  value of corrected mortality, X  =  livability of the control, Y  =  livability of the treat). The LC_50_ value (median lethal concentration) was obtained using probit analysis of the concentration-dependent mortality data by software DPS v7.05 for Window XP [31]. The biological data against *S. exigua* and *P. xylostella* in laboratory are listed in Table 1 and Table 2.

**Table 1:** Insecticidal Activities against Beet Armyworm.

Insecticides	Regression Equation	LC_50_ mg·L^−1^	R^b^	95% confidence limits
NK-17	0.92±0.07	3.38	0.9915	2.68–4.25
Hexaflumuron^a^	1.01±0.07	6.54	0.9924	5.45–7.84
chlorfluazuron^a^	0.93±0.04	9.09	0.9974	8.20–10.07

a hexaflumuron and chlorfluazuron were the main benzoylphenylurea chitin biosynthesis inhibitors widely used in vegetable in China as contrast pesticides. ^b^ R, correlative coefficient, its values are closer to 1 shows that the design and process of trial were better.

**Table 2:** Insecticidal Activities against Diamondback Moth.

Insecticides	Regression Equation	LC_50_ mg·L^−1^	R^b^	95% confidence limits
NK-17	1.34±0.09	26.73	0.9929	22.73–31.44
Hexaflumuron^a^	1.14±0.10	36.23	0.9883	29.99–43.77
chlorfluazuron^a^	1.23±0.10	50.80	0.9900	42.91–60.15

a hexaflumuron and chlorfluazuron were the main benzoylphenylurea chitin biosynthesis inhibitors widely used in vegetable in China as contrast pesticides. ^b^ R, correlative coefficient, its values are closer to 1 shows that the design and process of trial were better.

### Bioassay against *M. separate* in laboratory

The insecticidal activities of **NK-17** and the contrast hexaflumuron against *M*. *separata* were evaluated by foliar application according to the reported procedure [28]–[29]. For the foliar armyworm tests, individual corn leaves were placed on moistened pieces of filter paper in Petri dishes. The leaves were then sprayed with the test solution and allowed to dry. The dishes were infested with 10 fourth-instar oriental armyworm larvae. Percentage mortalities were evaluated 96 h after treatment. Each treatment was performed three times. The biological data in Table 3 was the average value of the three tested values.

**Table 3:** Insecticidal Activities against Oriental Armyworm.

Insecticides	Regression Equation	LC_50_ mg·L^−1^	R^b^	95% confidence limits
NK-17	Y = 9.7901+2.6968x	0.017	0.9850	0.011–0.025
Hexaflumuron^a^	Y = 6.4272+2.8013x	0.31	0.9949	0.24∼0.40

a Hexaflumuron was a main benzoylphenylurea chitin biosynthesis inhibitor widely used in vegetable in China as contrast pesticides. ^b^ R, correlative coefficient, its values are closer to 1 shows that the design and process of trial were better.

### Evaluation of insecticidal activities against *S. exigua* and *P. xylostella* in field

Field trials were obtain permits by Nankai University, and carried out between June and September during the 2011 and 2012 cropping seasons, in the Zhangjiawo vegetable production area, Xiqing district, Tianjin City, China (soil organic matter content of 2%, 42% sand, 30% silt and 28% clay, areas are public) and in Daqiao village, Yuhua district, Changsha City, Hunan Province, China (the soil: red earth, soil organic matter content of 4%, PH: 6.5, areas are public). The selected field sites are two of the most primary crop and vegetable production area in china and represent domain properties of north and south respectively. Because of consecutive cultivation and less effective rotation, the occurrence of the *S. exigua* and *P. xylostella* damages was very severe in these regions during the trials. Based on crop nutritional requirements, the field received a broadcast application of 200 kg ha^−1^ of 15N-10P-25K as starter fertilizer.

The treat plots of the beets were designed in the random block array. Moreover, each treat had four replicates in *S. exigua* field trials. According to the results of bioassay in the laboratory, 5% hexaflumuron EC at a dosage of 60 g active ingredient (a.i.) ha^−1^ (IV) was applied as a reference treatment; 5% **NK-17** EC was applied at a series of dosage of 15 g a.i. ha^−1^ (I), 30 g a.i. ha^−1^ (II) and 60 g a.i. ha^−1^ (III) in the Tianjin and Changsha trial fields. A nontreated control was also included and had four replicates. The *P. xylostella* field trials scheme was approximately same as that of *S. exigua* except for the contrast agent and the crops replaced by 5% chlorfluazuron EC (V) on the cabbages respectively. We investigate and counted the insect numbers before spraying pesticides, 1 day, 3 days, 7 days and 14 days after applying pesticides in the all treat plots.

The data expressed as percentages were arcsine transformed to homogenize variances before analysis, and then the effects of different spraying treatments were examined using analysis of variance (ANOVA) and when the F test was significant at P<0.05, treatment means were compared using the Duncan's new multiple range (DPS, v7.05 for Windows) [32].

### Action mechanism study of NK-17

#### Bioassay against B. germanica in vivo

Insecticidal activity of **NK-17** against cockroach (*B. germanica*) fifth-instar nymphs was assayed and compared with glibenclamide and diflubenzuron on the basis of the reported method [22] with some minor modifications. The aim of the assay was to determine the activity order of the three compounds, which would be used to compare with the order of bioactivity *(SUR binding affinity) in vitro*. A series of dilutions of each test compound were prepared in dimethylsulfoxide (DMSO): ethanol (1:1) according to wt/vol and expressed as µg/µL. The test was carried out by delivering a drop of test compound (about 1.0 µL) to the foregoing three abdominal sternites of nymphs of cockroach using a 5 µL Hamilton gas-tight syringe (Sigma-Aldrich). Seven concentrations were chosen to give the mortality percentage between 0% and 100%. Three replicates (10 nymphs per replicate) were performed for each concentration. Control cockroaches received only solvent mixture. Mortalities were recorded after 72 h. Cockroaches that could not respond to touch or always remained ventral side up when turning were considered dead. The molting symptoms was also observed and recorded in each assay.

#### Preparation of SUR

The integuments of cockroach were cut into small pieces and homogenized using a precisely fitted glass–glass tissue homogenizer in isotonic MES–sucrose buffer (10 mM MES containing 250 mM sucrose and 2.5 mM MgSO_4_, adjusted to pH 6.6 with NaOH). The homogenate was centrifuged for 15 min at 1000 g, and then the supernatant was further centrifuged at 10,000 g for 20 min, and the precipitate was obtained and suspended in the same buffer as the preparation of SUR [28].

#### NK-17 binding to SUR

To determine the superior concentration for the fluorescence probe, some total volume of 3000 µL reaction solutions were prepared by adding 100 µL SUR preparation prepared above to the proper quantity of MES–sucrose buffer containing different concentrations of N-phenyl-1-naphthylamine (1-NPN). Subsequently, ligands such as glibenclamide, diflubenzuron and **NK-17** in different concentrations was respectively added to the mixture containing SUR, buffer and 1-NPN, and then the mixture was incubated for 1 h at room temperature. The binding affinity was assayed by FP.

#### The FP assay

The value of FP was measured using Cary Eclipse Fluorescence Spectrofluorimeter (Agilent). The emission wavelength was emitted at 410 nm (with a slit width of 10 nm) and the excitation wavelength was emitted at 337 nm (with a slit width of 10 nm). Polarization is expressed as:
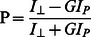



The *I_⊥_* and *I_∥_* represent the vertically and horizontally polarized emission intensities, respectively, while polarized excitation light is vertical. The G factor is the instrumental effects and can be expressed as:
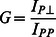



The *I_∥⊥_* and *I_∥∥_* represent the vertically and horizontally polarized emission intensities, respectively, while polarized excitation light is horizontal. The polarization of each sample was obtained from an average of 6 measurements each of *I_⊥_* and *I_∥_*, and five values of such a set of averages were collected.

## Results and Discussion

### Effects of insecticides against *S. exigua, P. xylostella* and *M. separate* in laboratory

**Figure 2:**
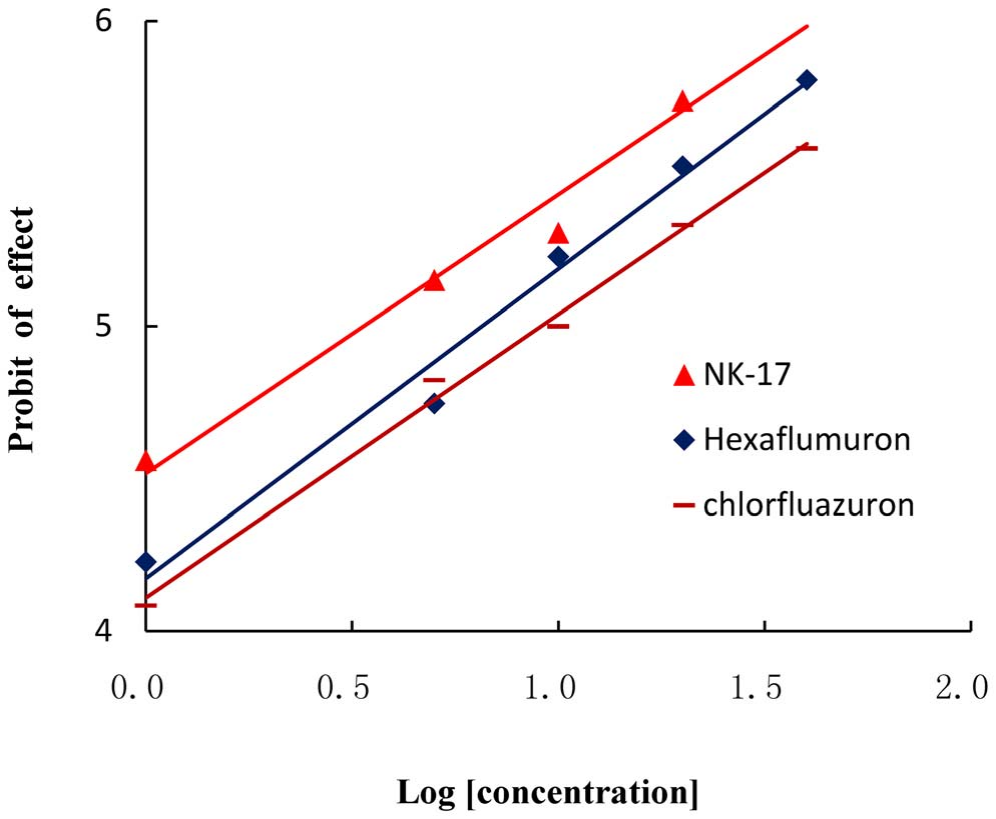
The concentration-effect curve of NK-17, Hexaflumuron and Chlorfluazuron. The Y-axis is the probit of mortality of Beet Armyworm, *S. exigua*, The X-axis is the logarithm value of the concentration of the insecticides, at last, the nymphs were assayed by using of leaves-dip method.

The concentration-effect curves of the bioassay results of **NK-17** and contrast compounds against *S. exigua* are presented in Figure 2. The LC_50_ values and the slope± SEM of **NK-17** were calculated according to the curve and are shown in Table 1. The LC_50_ (95% confidence limits) value of **NK-17** against *S. exigua* was 3.38 mg L^−1^ (2.68-4.25 mg L^−1^), whereas the LC_50_ (95% confidence limits) values of hexaflumuron and chlorfluazuron were 6.54 mg L^−1^ (5.45-7.84 mg L^−1^) and 9.09 mg L^−1^ (8.20-10.07 mg L^−1^), respectively. Hence, the toxicity of **NK-17** was 1.93 times and 2.69 times respectively those of hexaflumuron and chlorfluazuron.

**Figure 3:**
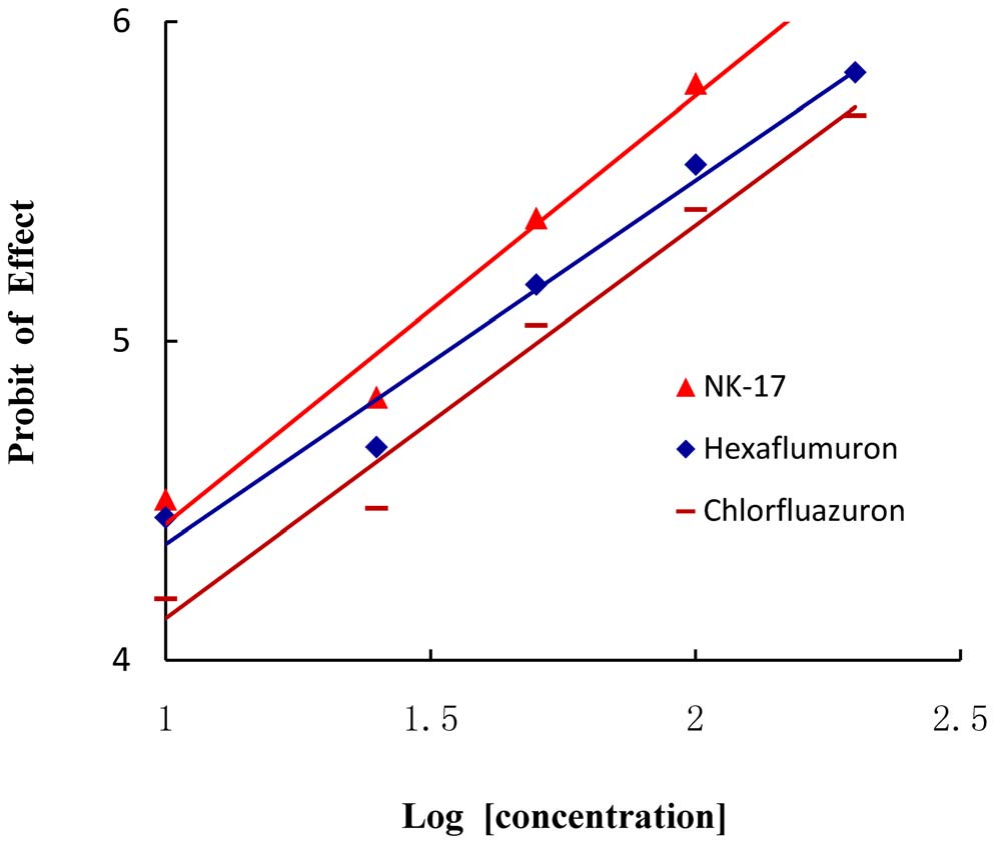
The concentration-effect curve of NK-17, Hexaflumuron and Chlorfluazuron. The Y-axis is the probit of mortality of Diamondback Moth, *P. xylostella,* accordingly, The X-axis is the logarithm value of the concentration of the insecticides, at last, the nymphs were assayed by using of leaves-dip method.

Similarly, in Table 2 and 3, the LC_50_ (95% confidence limits) value of **NK-17** against *P. xylostella* was 26.73 mg L^−1^ (22.73-31.44 mg L^−1^), and the toxicity of **NK-17** was 1.36 times and 1.90 times of hexaflumuron and chlorfluazuron (Figure 3). The LC_50_ values and the toxicity regression equations of **NK-17** were calculated according to the curve and are shown in Table 3. The LC_50_ (95% confidence limits) value of **NK-17** against *M. separate* was 0.017 mg L^−1^ (0.011-0.025 mg L^−1^), and the toxicity of **NK-17** was 18.24 times of hexaflumuron.

As **NK-17** had definitely stronger toxicity than hexaflumuron and chlorfluazuron, faster action of **NK-17** than those compounds could be observed from the mortality of *S. exigua, P. xylostella* and *M. separate* at 48 h. More importantly, the investigation of toxic symptoms showed that the action mode of **NK-17** is similar, if not identical to hexaflumuron and chlorfluazuron. The treated larvae displayed several obvious characters of moulting defects, which were a double head capsule caused by the disability to shed the old cutile, and incomplete ecdysis caused by loss of haemolymph at the joint of the new head capsule and the thoracic segments, in that the typical molting problems were observed, and some dying insects still attached to the old cuticles.

### Evaluation of insecticidal activities against *S. exigua* in field

**Figure 4:**
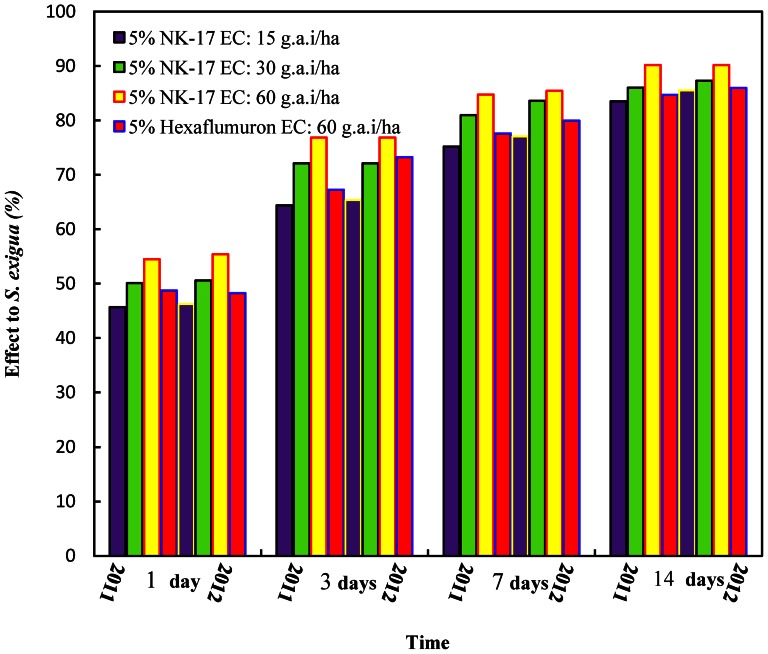
Evaluation of 5% NK-17 EC against *S. exigua*. The trials was carried out in the cabbages in the Zhangjiawo vegetable production area, Xiqing district, Tianjin City, China, in 2011 and 2012. The 5%NK-17 EC had the better performance on controlling *S. exigua* in the cabbage field than the commercial insecticides 5% Hexaflumuron.

Field trials of insecticidal activities against *S. exigua* were carried out in Tianjin and Changsha during the 2011 and 2012 cropping seasons. The two year results of the field trials on *S. exigua* in Tianjin were given in Figure 4. It showed that 5% **NK-17** EC at the dose of 60 g a.i. ha^−1^ had better control effect than the comparison 5% hexaflumuron EC at the same dosage. The significance of difference was analyzed using Duncan's new multiple range. The effect data of the trial of Tianjin were indicated in 2011 (Table 4), 1 day after, the control efficiency of 5% **NK-17** EC at 60 g a.i. ha^−1^ was 54.50% comparing to 48.75% of 5% hexaflumuron EC at the same conditions. Therefore, The speed of action of 5% **NK-17** EC was faster than 5% hexaflumuron EC. 14 days after, the control efficiency of 5% **NK-17** EC at 60 g a.i. ha^−1^ was 90.19% comparing to 84.71% of 5% hexaflumuron EC. The data of control efficiency in 2012 (Table 5) were similar, if not identical, to that in 2011, for example, the control efficiency of 5% **NK-17** EC at 60 g a.i. ha^−1^ achieved to 90.16% in 14 days comparing to 85.97% of 5% hexaflumuron EC.

**Table 4:** Effect of insecticidal progam on *S. exigua* in Tianjin City in 2011.

	1 day after treated^b^			3 day after treated			7 day after treated			14day after treated		
Treats^a^	Effect(%)	Significance of difference^ c^		Effect(%)	Significance of difference		Effect(%)	Significance of difference		Effect(%)	Significance of difference	
		0.05	0.01		0.05	0.01		0.05	0.01		0.05	0.01
I	45.69	b	A	64.41	b	A	75.18	b	A	83.52	b	A
II	50.09	ab	A	72.11	ab	A	80.95	ab	A	85.99	b	B
III	54.50	a	A	76.83	a	A	84.74	a	A	90.19	a	B
IV	48.75	ab	A	67.26	ab	A	77.61	b	A	84.71	b	B
control	-	-	-	-	-	-	-	-	-	-	-	-

a I: 5% NK-17 EC, 15 g a.i. ha^−1^, II: 5% NK-17 EC, 30 g a.i. ha^−1^, III: 5% NK-17 EC, 60 g a.i. ha^−1^ NK-17; IV: 5% hexaflumuron EC, 60 g a.i. ha^−1^. ^b^days after treats: the number of each plot were investigated, days after spraying insecticides.^ c^: the significance of difference among the all treats were computed using Duncan's new multiple range to give the level of difference in p = 0.05 and p = 0.01.

**Table 5:** Effect of insecticidal progam on *S. exigua* in Tianjin City in 2012.

	1 day after treated^b^			3 day after treated			7day after treated			14 day after treated		
Treats^a^	Effect(%)	Significance of difference^c^		Effect(%)	Significance of difference		Effect(%)	Significance of difference		Effect(%)	Significance of difference	
		0.05	0.01		0.05	0.01		0.05	0.01		0.05	0.01
I	46.23	b	A	65.37	b	B	77.02	b	A	85.49	b	B
II	50.56	ab	A	72.10	a	AB	83.57	a	A	87.30	ab	AB
III	55.40	a	A	76.83	a	A	85.42	a	A	90.16	a	A
IV	48.24	ab	A	73.25	a	AB	79.94	ab	A	85.97	b	AB
control	-	-	-	-	-	-	-	-	-	-	-	-

a I: 5% NK-17 EC, 15 g a.i. ha^−1^, II: 5% NK-17 EC, 30 g a.i. ha^−1^, III: 5% NK-17 EC, 60 g a.i. ha^−1^ NK-17; IV: 5% hexaflumuron EC, 60 g a.i. ha^−1^. ^b^days after treats: the number of each plot were investigated, days after spraying insecticides.^ c^: the significance of difference among the all treats were computed using Duncan's new multiple range to give the level of difference in p = 0.05 and p = 0.01.

**Figure 5:**
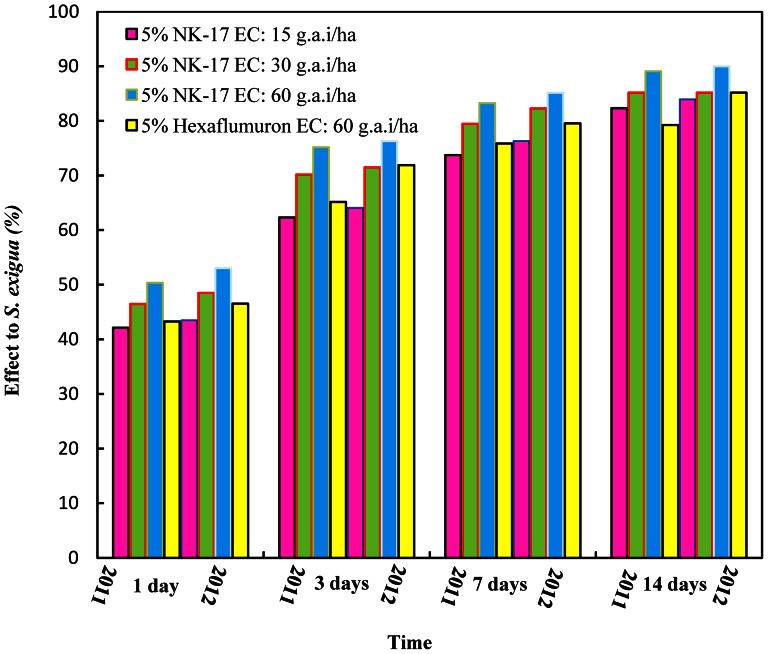
Evaluation of 5% NK-17 EC against *S. exigua*. The trials was carried out in the cabbages in Daqiao village Yuhua district, Changsha City, Hunan Province, China, in 2011 and 2012. The 5% NK-17 EC had the better performance on controlling *S. exigua* in the cabbage field than the commercial insecticides 5% Hexaflumuron.

The control efficiency of the trial in Changsha was exhibited in Figure 5, and the result was almost same as Tianjin, so did the data of the trial in Changsha (Table 6). 1 day after, the control efficiency of 5% **NK-17** EC at 60 g a.i. ha^−1^ was 50.31% comparing to 43.28% of 5% hexaflumuron EC. Therefore, the speed of action of 5% **NK-17** EC was faster than 5% hexaflumuron EC. 14 days after, the control efficiency of 5% **NK-17** EC at 60 g a.i. ha^−1^ was 89.06% comparing to 79.26% of 5% hexaflumuron EC. The data of control efficiency in 2012 (Table 7) were similar, if not identical, to that in 2011, for example, the control efficiency of 5% **NK-17** EC at 60 g a.i. ha^−1^ achieved to 89.97% in 14 days comparing to 85.14% of 5% Hexaflumuron EC.

**Table 6:** Effect of insecticidal progam on *S. exigua* Changsha City, Hunan Province in 2011.

	1 day after treated^b^			3 day after treated			7 day after treated			14day after treated		
Treats^a^	Effect(%)	Significance of differencec^c^		Effect(%)	Significance of difference		Effect(%)	Significance of difference		Effect(%)	Significance of difference	
		0.05	0.01		0.05	0.01		0.05	0.01		0.05	0.01
I	42.13	a	A	62.32	b	B	73.73	b	A	82.30	bc	B
II	46.44	a	A	70.18	ab	AB	79.47	ab	A	85.14	ab	AB
III	50.31	a	A	75.15	a	A	83.23	a	A	89.06	a	A
IV	43.28	a	A	65.15	b	AB	75.86	b	A	79.26	c	B
control	-	-	-	-	-	-	-	-	-	-	-	-

a I: 5% NK-17 EC, 15 g a.i. ha^−1^, II: 5% NK-17 EC, 30 g a.i. ha^−1^, III: 5% NK-17 EC, 60 g a.i. ha^−1^ NK-17; IV: 5% hexaflumuron EC, 60 g a.i. ha^−1^. ^b^days after treats: the number of each plot were investigated, days after spraying insecticides.^ c^: the significance of difference among the all treats were computed using Duncan's new multiple range to give the level of difference in p = 0.05 and p = 0.01.

**Table 7:** Effect of insecticidal progam on *S. exigua* Changsha City, Hunan Province in 2012.

	1 day after treated^b^			3 day after treated			7day after treated			14 day after treated		
Treats^a^	Effect(%)	Significance of difference^c^		Effect(%)	Significance of difference		Effect(%)	Significance of difference		Effect(%)	Significance of difference	
		0.05	0.01		0.05	0.01		0.05	0.01		0.05	0.01
I	43.49	b	A	64.07	b	B	76.34	b	A	83.92	b	B
II	48.49	ab	A	71.49	a	AB	82.28	ab	A	85.14	b	AB
III	53.01	a	A	76.29	a	A	85.11	a	A	89.97	a	A
IV	46.50	ab	A	71.88	a	AB	79.55	ab	A	85.14	b	AB
control	-	-	-	-	-	-	-	-	-	-	-	-

a I: 5% NK-17 EC, 15 g a.i. ha^−1^, II: 5% NK-17 EC, 30 g a.i. ha^−1^, III: 5% NK-17 EC, 60 g a.i. ha^−1^ NK-17; IV: 5% hexaflumuron EC, 60 g a.i. ha^−1^. ^b^days after treats: the number of each plot were investigated, days after spraying insecticides.^ c^: the significance of difference among the all treats were computed using Duncan's new multiple range to give the level of difference in p = 0.05 and p = 0.01.

Hence, it was suggested that 5% **NK-17** EC was a promising insecticide as alternative to the high toxic pesticides against *S. exigua* according to the two years and two sites trials.

### Evaluation of insecticidal activities against *P. xylostella* in field

**Figure 6:**
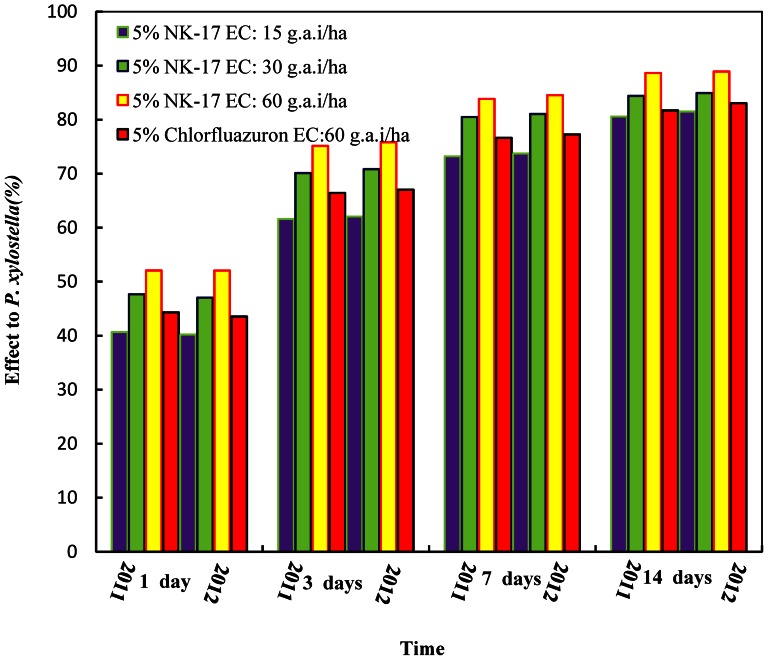
Evaluation of 5% NK-17 EC against *P. xylostella*. The trials was carried out in the Zhangjiawo vegetable production area, Xiqing district, Tianjin City, China, in 2011 and 2012. The 5% NK-17 EC had the better performance on controlling *P. xylostella* in the cabbage field than the commercial insecticides 5% chlorfluazuron.

Firstly, the results of the trials in Tianjin including 2011 and 2012 controlling *P. xylostella* were given at 1 day, 3 days, 7 days, and 14 days respectively in Figure 6. It showed obviously that 5% **NK-17** EC had better control effect than the comparison 5% Chlorfluazuron EC at the all same conditions. The significance of difference was analyzed using Duncan's new multiple range. The effect data of the trial of Tianjin were indicated in 2011 (Table 8), 1 day after, the control efficiency of 5% **NK-17** EC at maximum dosage was 52.07% comparing to 44.31% of 5% Chlorfluazuron EC at the same conditions. Therefore, the speed of action of 5% **NK-17** EC was faster than 5% Chlorfluazuron EC. 14 days after, the control efficiency of 5% **NK-17** EC at maximum dosage was 88.64% comparing to 81.69% of 5% Chlorfluazuron EC. The data of control efficiency in 2012 (Table 9) were about identical to that in 2011, one of which indicated the control efficiency of 5% **NK-17** EC at the same maximum dosage achieving to 88.91% in 14 days comparing to 83.03% of 5% Chlorfluazuron EC.

**Table 8:** Effect of insecticidal progam on *P. xylostella* in Tianjin City in 2011.

	1 day after treated^b^			3 day after treated			7 day after treated			14day after treated		
Treats^a^	Effect(%)	Significance of difference^c^		Effect(%)	Significance of difference		Effect(%)	Significance of difference		Effect(%)	Significance of difference	
		0.05	0.01		0.05	0.01		0.05	0.01		0.05	0.01
I	40.68	a	A	61.60	b	B	73.20	b	B	80.57	b	B
II	47.65	a	A	70.12	a	AB	80.50	a	AB	84.37	b	AB
III	52.07	a	A	75.15	a	A	83.82	a	A	88.64	a	A
V	44.31	a	A	66.43	ab	AB	76.64	ab	AB	81.69	b	B
control	-	-	-	-	-	-	-	-	-	-	-	-

a I: 5% NK-17 EC, 15 g a.i. ha^−1^, II: 5% NK-17 EC, 30 g a.i. ha^−1^, III: 5% NK-17 EC, 60 g a.i. ha^−1^ NK-17; V: 5% Chlorfluazuron EC, 60 g a.i. ha^−1^. ^b^days after treats: the number of each plot were investigated, days after spraying insecticides.^ c^: the significance of difference among the all treats were computed using Duncan's new multiple range to give the level of difference in p = 0.05 and p = 0.01.

**Table 9:** Effect of insecticidal progam on *P. xylostella* in Tianjin City in 2012.

	1 day after treated^b^			3 day after treated				7day after treated						14 day after treated					
Treats^a^	Effect(%)	Significance of difference^c^		Effect(%)	Significance of difference			Effect(%)		Significance of difference				Effect(%)		Significance of difference			
		0.05	0.01		0.05	0.01				0.05		0.01				0.05		0.01	
I	40.21	a	A	62.04	b	B		73.72		b		B		81.46		b		B	
II	47.01	a	A	70.84	ab	AB	81.05		a		AB		84.92		ab		AB		
III	52.05	a	A	75.84	a	A		84.52		a		A		88.91		a		A	
V	43.53	a	A	67.03	ab	AB		77.28		ab		AB		83.03		b		AB	
control	-	-	-	-	-	-		-		-		-		-		-		-	

a I: 5% NK-17 EC, 15 g a.i. ha^−1^, II: 5% NK-17 EC, 30 g a.i. ha^−1^, III: 5% NK-17 EC, 60 g a.i. ha^−1^ NK-17; V: 5% Chlorfluazuron EC, 60 g a.i. ha^−1^. ^b^days after treats: the number of each plot were investigated, days after spraying insecticides.^ c^: the significance of difference among the all treats were computed using Duncan's new multiple range to give the level of difference in p = 0.05 and p = 0.01.

**Figure 7:**
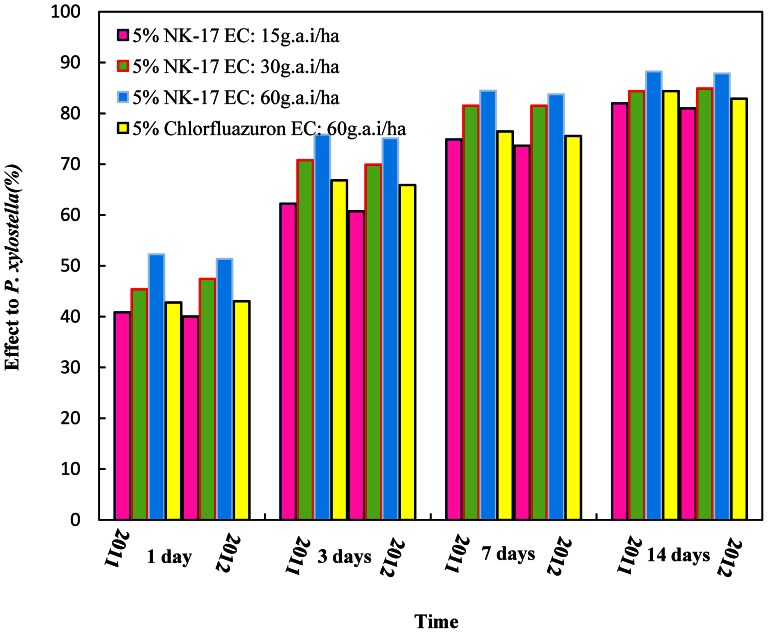
Evaluation of 5% NK-17 EC against *P. xylostella*. The trials was carried out in the cabbages in Daqiao village Yuhua district, Changsha City, Hunan Province, China, in 2011 and 2012. The 5% NK-17 EC had the better performance on controlling *P. xylostella* in the cabbage field than the commercial insecticides 5% chlorfluazuron.

The results of the trial in Changsha were showed in Figure 7, and the evaluation of the efficiency was almost same as Tianjin, so did the data of the trial in Changsha in 2011 (Table 10). 1 day after, the control efficiency of 5% **NK-17** EC at the same maximum dosage was 51.35% comparing to 43.04% of 5% Chlorfluazuron EC. Therefore, the fast action of 5% **NK-17** EC was suprior than 5% Chlorfluazuron EC. 14 days after, the control efficiency of 5% **NK-17** EC at 60 g a.i. ha^−1^ was 87.85% comparing to 82.88% of 5% Chlorfluazuron EC. The data of control efficiency in 2012 (Table 11) were similar, if not identical, to that in 2011, one of which showed the control efficiency of 5% **NK-17** EC at the maximum dosage achieving to 88.22% in 14 days comparing to 84.37% of 5% Chlorfluazuron EC on the same conditions.

**Table 10:** Effect of insecticidal progam on *P. xylostella* Changsha City, Hunan Province in 2011.

	1 day after treated			3 day after treated			7 day after treated			14day after treated		
Treats	Effect(%)	Significance of difference		Effect(%)	Significance of difference		Effect(%)	Significance of difference		Effect(%)	Significance of difference	
		0.05	0.01		0.05	0.01		0.05	0.01		0.05	0.01
I	40.03	a	A	60.75	b	B	73.63	c	A	81.01	c	B
II	47.39	a	A	69.93	a	AB	81.52	ab	A	84.87	ab	AB
III	51.35	a	A	75.11	a	A	83.74	a	A	87.85	a	A
V	43.04	a	A	65.90	ab	AB	75.55	bc	A	82.88	bc	AB
control	-	-	-	-	-	-	-	-	-	-	-	-

a I: 5% NK-17 EC, 15 g a.i. ha-^1^, II: 5% NK-17 EC, 30 g a.i. ha^−1^, III: 5% NK-17 EC, 60 g a.i. ha^−1^ NK-17; V: 5% Chlorfluazuron EC, 60 g a.i. ha^−1^. ^b^days after treats: the number of each plot were investigated, days after spraying insecticides.^ c^: the significance of difference among the all treats were computed using Duncan's new multiple range to give the level of difference in p = 0.05 and p = 0.01.

**Table 11:** Effect of insecticidal progam on *P. xylostella* Changsha City, Hunan Province in 2012.

	1 day after treated^b^			3 day after treated			7day after treated			14 day after treated		
Treats^a^	Effect(%)	Significance of difference^c^		Effect(%)	Significance of difference		Effect(%)	Significance of difference		Effect(%)	Significance of difference	
		0.05	0.01		0.05	0.01		0.05	0.01		0.05	0.01
I	40.86	a	A	62.23	b	A	74.87	b	A	82.00	b	B
II	45.39	a	A	70.80	ab	A	81.53	ab	A	84.37	ab	AB
III	52.26	a	A	75.82	a	A	84.45	a	A	88.22	a	A
V	42.79	a	A	66.83	ab	A	76.45	b	A	84.37	ab	AB
control	-	-	-	-	-	-	-	-	-	-	-	-

a I: 5% NK-17 EC, 15 g a.i. ha^−1^, II: 5% NK-17 EC, 30 g a.i. ha^−1^, III: 5% NK-17 EC, 60 g a.i. ha^−1^ NK-17; V: 5% Chlorfluazuron EC, 60 g a.i. ha^−1^. ^b^days after treats: the number of each plot were investigated, days after spraying insecticides.^ c^: the significance of difference among the all treats were computed using Duncan's new multiple range to give the level of difference in p = 0.05 and p = 0.01.

Hence, it was suggested that 5% **NK-17** EC was a promising insecticide as alternative to the high toxic pesticides against *P. xylostella* depending on the results of the two years and two sites trials.

### Action mechanism study of NK-17

#### Effects of NK-17 against *B. germanica* in laboratory

**Figure 8:**
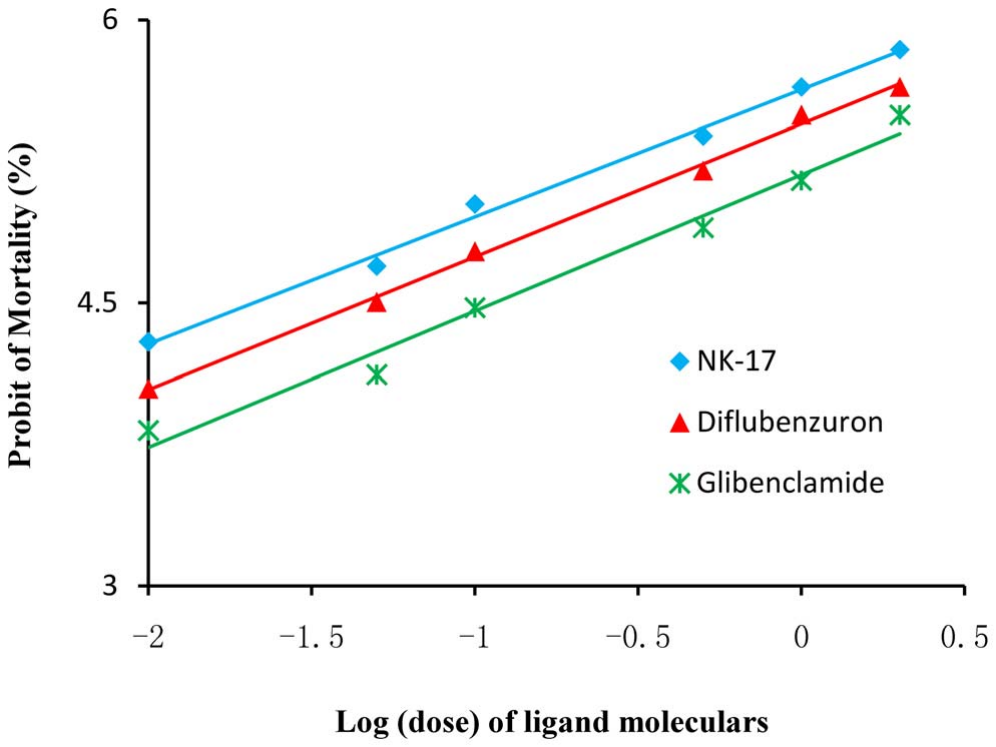
The concentration-effect curve of NK-17, Diflubenzuron and Glibenclamide. The Y-axis is the probit of mortality of cockroach, *B. germanica*, The X-axis is the logarithm value of the concentration of the insecticides, at last, the nymphs were assayed by using topical application technique. By passing of Probit analysis for NK-17: Slope±SEM = 0.67±0.03, the LD_50_ (95% confidence limits) value of NK-17 was 0.12 mg·L^−1^ (0.10–0.13 mg·L^−1^), whereas the LD_50_ (95% confidence limits) values of diflubenzuron and glibenclamide were 0.23 mg·L^−1^ (0.21–0.25 mg·L^−1^) and 0.56 mg·L^−1^ (0.42–0.75 mg·L^−1^), respectively.

Insecticidal activity of **NK-17**, diflubenzuron and glibenclamide against *B. germanica in vivo* was firstly assayed. The aim of the assay was to determine the activity order of the three compounds, which would be used to compare with the order of bioactivity *in vitro* (SUR binding affinity). According to the concentration-effect curves of the bioassay results presented in Figure 8, the order of toxicity to *B. germanica* was **NK-17** > diflubenzuron > glibenclamide according to the LD_50_ of 0.12 mg L^−1^, 0.23 mg L^−1^, 0.56 mg L^−1^, respectively. Moreover, the molting symptom of **NK-17** was identical to diflubenzuron and glibenclamide, which suggested **NK-17** may have the same action mechanism as diflubenzuron and glibenclamide.

#### Affinity of NK-17 binding to SUR

As previously mentioned, by utilizing the isotope labeling technology, diflubenzuron was testified to act on the same target site of SUR proteins as glibenclamide [22]. In this paper, we would like to adopt fluorescence polarization (FP) method to ascertain if **NK-17** could also bind in the same site of SUR.

The FP assay is based on the high affinity binding of the fluorescence probe N-phenyl -1-naphthylamine (1-NPN) to a specific site of SUR. After absorbing polarized light, free 1-NPN of relatively small molecular weight emits light in all directions due to the fast tumbling rate, resulting in low polarization. Binding to the target protein SUR, the 1-NPN molecules rotate slower due to the larger combined molecular size of the complex. Consequently, they emit radiation in the same direction as that of the incident light, and exhibit higher polarization. When some compounds can displace the 1-NPN molecules from SUR, the disruption of the binding between the 1-NPN molecules and the protein can be identified by decreased polarization.

First of our work was to test and verify the feasibility of this FP assay by the competitive displacement of 1-NPN with glibenclamide and diflubenzuron from 1-NPN-SUR. The SUR proteins of *B. germanica* were prepared by the reported method with some minor modifications. However, the concentration of the SUR cannot be accurately measured without its crystal structure, because its gene has never decoded so far. Furthermore, the vesicles containing the SUR were not stable enough for the receptor to bind to the fluorescence probe after a long time. Therefore, the binding affinity of **NK-17**, diflubenzuron and glibenclamide with SUR were compared within the same set of experiments with the same vesicle preparation, and over 5 replicates must be carried out to further minimize the error.

**Figure 9:**
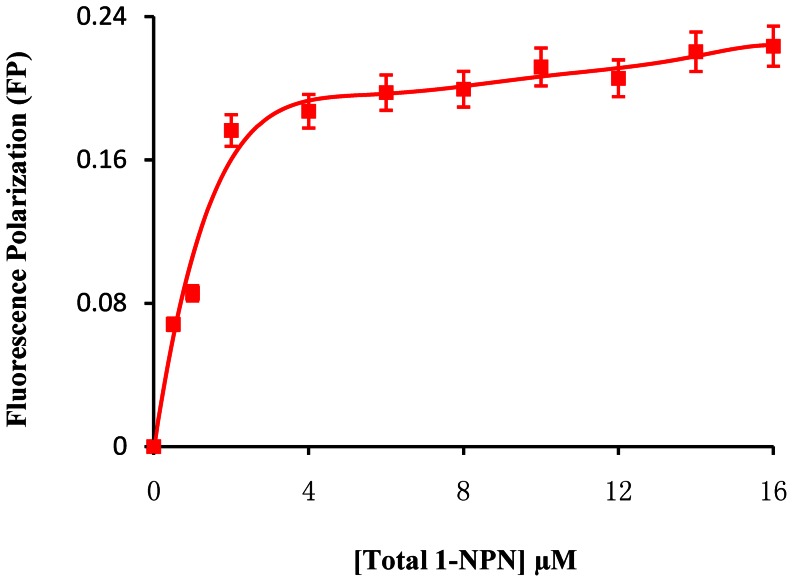
Binding of 1-NPN ligand to the sSUR. Binding of 1-NPN to the SUR protein in the MES-sucrose. As the concentration of the fluorescence probe 1-NPN was gradually elevated, the value of fluorescence polarization was according improved in the origin. When the concentration was increase to 3 µM, the value of FP began to keep a balance. That indicates that the binding of 1-NPN to SUR was saturated, therefore, 3 µM was used as the standand concentration in investing the comparative affinity to other ligands in the experiment.

Because of previous discussion, we had to measure the affinity of the probe binding to SUR to elucidate the dose of the probe very time (Figure 9). At beginning, the FP was quickly raised by increasing the dosage of the probe to indicate the all probe binding to the SUR in order to find the most proper concentration of the probe [33].

**Figure 10:**
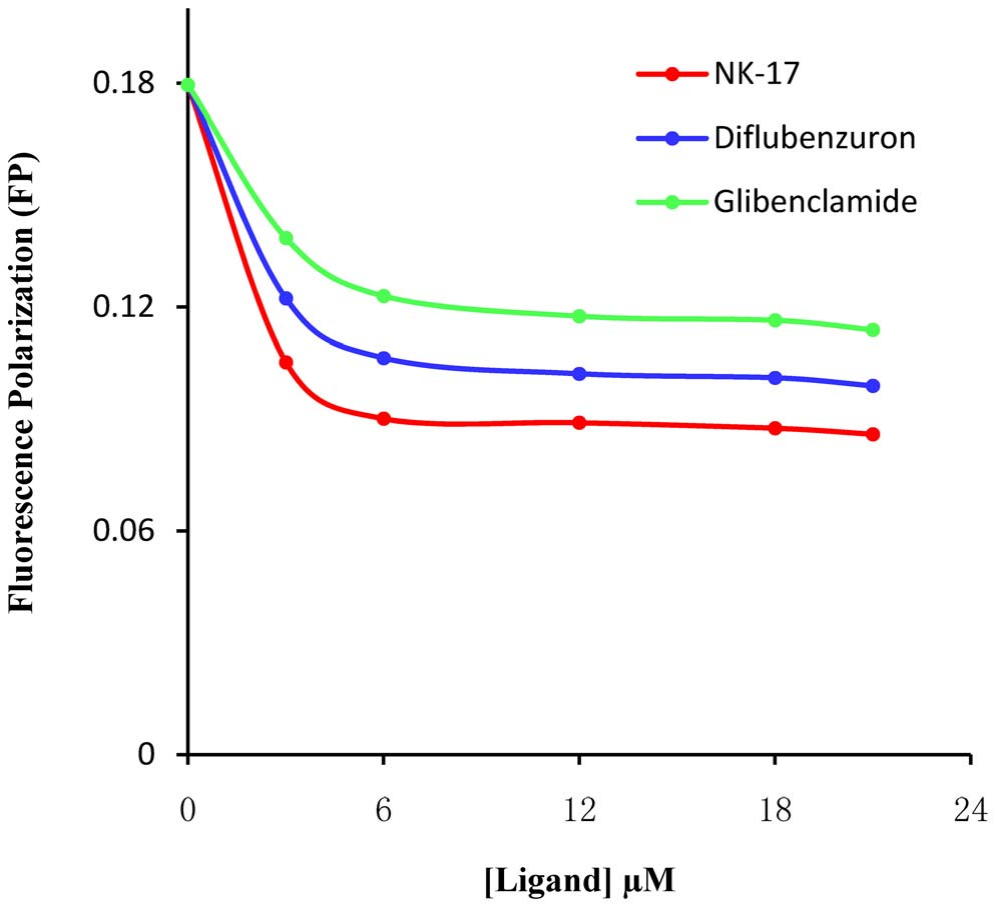
Binding of NK-17, Glibenclamide and diflubenzuron to SURs. Binding reactions were incubated for 1 h at room temperature. All measurements were done in tetraplicate; data are the average of the all repeat experiments. The affinity was assayed using fluorescence polarization. The lower FP indicates the more intensive receptor-ligand binding affinity.

With the most proper concentration of the 1-NPN-SUR, **NK-17**, diflubenzuron and glibenclamide as competitive ligands binding to the SUR were assayed by FP (Figure 10). According to the affinity result of the three compounds, the order was **NK-17** > diflubenzuron > glibenclamide, which was consistent with the bioactivity against *B. germanica in vivo*.

## Conclusions

Our research on insecticidal activities of **NK-17** in laboratory and in field (two years, two trial sites) showed that the toxicity of **NK-17** against *S. exigua* was 1.93 times and 2.69 times respectively those of hexaflumuron and chlorfluazuron, and the toxicity of **NK-17** against *P. xylostella* was 1.36 times and 1.90 times respectively those of hexaflumuron and chlorfluazuron, and the toxicity of **NK-17** against *M. separate* was 18.24 times those of hexaflumuron in laboratory. 5% **NK-17** EC at 60 g a.i. ha^−1^ can control *S. exigua* with the best control efficiency of about 89% in Changsha and Tianjin in field, which suggested that 5% **NK-17** EC a promising insecticide as alternative to the high toxic insecticide against *S. exigua*. 5% **NK-17** EC at 60 g a.i. ha^−1^ can control *P. xylostella* with the best control efficiency of over 88% in Changsha and Tianjin, which suggested that 5% **NK-17** EC a promising insecticide as alternative to the high toxic pesticides against *P. xylostella*. Therefore, we conclude that **NK-17** is a very comprising insecticide candidate for controlling pests in crops, vegetables and fruits.

We explored the insecticidal mechanism of **NK-17** by utilizing fluorescence polarization method for the first time. **NK-17** could bind to SUR with stronger affinity comparing to DFB and glibenclamide, and the result can well explained that **NK-17** exhibited stronger toxicity against *B. germanica* than DFB and glibenclamide *in vivo*. It is suggested that **NK-17** may act on the site of SUR to inhibit the chitin synthesis in insect body.
